# Construction of High-Density Genetic Map and Mapping Quantitative Trait Loci for Growth Habit-Related Traits of Peanut (*Arachis hypogaea* L.)

**DOI:** 10.3389/fpls.2019.00745

**Published:** 2019-06-12

**Authors:** Li Li, Xinlei Yang, Shunli Cui, Xinhao Meng, Guojun Mu, Mingyu Hou, Meijing He, Hui Zhang, Lifeng Liu, Charles Y. Chen

**Affiliations:** ^1^North China Key Laboratory for Crop Germplasm Resources of Education Ministry, College of Agronomy, Hebei Agricultural University, Baoding, China; ^2^Department of Crop, Soil and Environmental Sciences, Auburn University, Auburn, AL, United States

**Keywords:** cultivated peanut (*Arachis hypogaea* L.), high-density genetic map, SLAF-seq, QTL mapping, plant growth habit

## Abstract

Plant growth habit is an important and complex agronomic trait and is associated with yield, disease resistance, and mechanized harvesting in peanuts. There are at least two distinct growth habits (erect and prostrate) and several intermediate forms existing in the peanut germplasm. A recombinant inbred line population containing 188 individuals was developed from a cross of “Jihua 5” and “M130” for genetically dissecting the architecture of the growth habit. A new high-density genetic linkage map was constructed by using specific locus amplified fragment sequencing technology. The map contains 2,808 single-nucleotide polymorphism markers distributed on 20 linkage groups with a total length of 1,308.20 cM and an average inter-marker distance of 0.47 cM. The quantitative trait locus (QTL) analysis of the growth habit-related traits was conducted based on phenotyping data from seven environments. A total of 39 QTLs for growth habit-related traits was detected on 10 chromosomes explaining 4.55–27.74% of the phenotypic variance, in which 6 QTLs were for lateral branch angle, 8 QTLs were for extent radius, 7 QTLs were for the index of plant type, 11 QTLs were for main stem height, and 7 QTLs were for lateral branch length. Among these QTLs, 12 were co-localized on chromosome B05 spanning an approximately 0.17 Mb physical interval in comparison with the allotetraploid reference genome of “Tifrunner.” Analysis of the co-localized genome region has shown that the putative genes are involved in light and hormones and will facilitate peanut growth habit molecular breeding and study of peanut domestication.

## Introduction

Peanut, or groundnut, is one of the most important oilseed crops and plays an important role in satisfying the demands for oil and protein throughout the world. The architecture of plants in nature is viewed as the vegetative and reproductive branching patterns, the relative arrangement of lateral organs, and differential lengths of the stem (Gallavotti, 2013). Plant architecture is associated with yield, disease resistance, and adaptation to mechanized harvesting. Donald (1968) coined the term “ideotype” which is described as optimum plant architecture. He defined the ideotype as plants that have comprehensive agronomic traits known to influence photosynthesis, growth habit, and grain yield (in cereals). In order to find the ideotype of peanut, many researchers have studied plant architecture including main stem height (MSH), first lateral branch length (LBL), and total branch number (Hake et al., 2017; Li Y. et al., 2017; Lv et al., 2018; Wang et al., 2018). There are at least two distinct growth habits (erect and prostrate) and several intermediate forms existing in the peanut germplasm (Coffelt, 1974). Erect genotypes have compact plant types and the pods are mainly concentrated at the bottom of plant. This type is most suitable for high-density planting. In contrasting, prostrate genotypes have larger spreading areas and the lateral branches of prostrate species creep along the ground, and so is better for pegging into soil. The branching habit trait was classified on a continuous scale from 1 (prostrate) to 6 (erect) according to the ratio of the length of the creeping to the length of the first lateral branch, and the angle between the main stem and the first lateral branch (Jiang et al., 2006). For domesticated peanut, plant types are readily classified into four types: prostrate, spreading, bunch, and erect (Kayam et al., 2017). The index of plant type (IOPT) (the ratio of the longest branch of the first pair lateral branches to the height of the main stem) is also used for quantifying the classification of peanut growth habit into three peanut plant types. The index for creeping plant type is around 2.0 and that of semi-creeping plant type is around 1.5. The erect plant type index is about 1.1–1.2 (Cao and Wang, 2013). Despite so many ways to define the growth habit, it is still difficult to distinguish those categories. Unlike other crops, peanuts bloom above ground and develop fruitage underground, so the growth pattern of lateral branches is an important characterization of peanut growth habit. Not only does it affect the peg penetration into the soil to form pods smoothly, but also closely relate to planting density. Hence, the angle between the erect main stem and the first lateral branch is becoming an important indicator of descriptive characteristics of plant architecture in peanut. In addition, MSH and the first LBL of peanut are important agronomic traits affecting growth habit and the number of the pods (Lan et al., 2011; Li Y. et al., 2017).

Peanut (*Arachis hypogaea* L., 2*n* = 4*x* = 40, genome AABB) probably derived from a hybridization event between two diploid species, *Arachis duranensis* (AA) and *Arachis ipaënsis* (BB) (Halward et al., 1992). The total genome size of AABB-type is around 2.7 GB, with an estimated repetitive content of 64% (Bertioli et al., 2016). A legacy of the “domestication bottleneck” is that cultivated peanut contains narrow genetic diversity, in which heritable variation is less than 13% (Varshney et al., 2009b). To date, there have been a large number of genetic maps constructed with various types of molecular markers, including restriction fragment length polymorphism (RFLP), amplified fragment length polymorphism (AFLP), cleaved amplified polymorphism sequences (CAPSs), and simple sequence repeat (SSR) markers. Recently, a large number of *AhMITE1* markers were developed from the whole-genome re-sequencing data in peanut, which can be used for trait mapping with a high degree of polymorphism (Gayathri et al., 2018). However, most of these maps are low-density genetic maps because of the low throughput of molecular markers. The low-density genetic maps have limited the efficiency and accuracy of quantitative trait locus (QTL) mapping. Therefore, high-density genetic maps are the foundation for mapping QTL, marker-assisted selection (MAS), and map-based cloning. With the advance of high-throughput sequencing technologies, several effective methods for obtaining thousands of single-nucleotide polymorphism (SNP) or insertion/deletions (InDel) markers at relatively low costs make it possible to construct a high-density genetic map, such as restriction site-associated sequencing (RAD-seq) (Miller et al., 2007), double digest RAD-seq (Peterson et al., 2012), and genotyping by sequencing (GBS) (Poland et al., 2012). Sun et al. (2013) developed a specific length amplified fragment sequencing (SLAF-seq) method to detect large numbers of SNPs. SLAF-seq is based on reduced representation library (RRL) and high-throughput sequencing, which involves fragment length selection but not through random interruption. Therefore, its repeatability and accuracy are better than RAD-seq and GBS (Qi et al., 2014; Xu Y. et al., 2015). By contrast, the method of SLAF-seq not only provides an economical and efficient approach to large-scale genotyping, but can also explore a large number of SNP and InDel markers at the same time. SLAF-seq technology has been used to construct high-density genetic maps for cotton (Keerio et al., 2018), cucumber (Xu X. et al., 2015), soybean (Li B. et al., 2017), and peanut (Hu et al., 2018). Moreover, many peanut SNP genetic linkage maps using different sequencing methods have been constructed. Zhou et al. (2014) and Han et al. (2018) developed genetic maps using ddRAD-seq and GBS, respectively. Wang et al. (2018) and Hu et al. (2018) detected a large number of SNPs through SLAF-seq and constructed a high-resolution map, respectively.

Peanuts are a dicotyledonous plant, and the molecular genetic basis of growth habit has not been clearly elucidated. In the early years, researchers analyzed the genetic regularity of peanut growth habit using Mendelian Law based on the different mode of inheritance between the runner and bunch types. Patel et al. (1936), Silvestre (1961), and Gan et al. (1984) indicated that growth habit was controlled by a single gene, but Hayes (1933) stated that two genes controlled the trait based on the separation ratio of 15:1 in the F_2_ progeny. Higgins (1938) demonstrated that the growth habit of peanut was a complicated trait with multiple genes involved. According to hybridization and reciprocal crosses, it was showed that nuclear and cytoplasmic interaction controls growth habit in peanuts (Ashri, 1964, 1968). On the contrary, Kayam et al. (2017) revealed that the branching habit was controlled by a single gene without cytoplasmic effect by reciprocal cross experiments. These different results are due to from different classification for plant type evaluation. In previous studies, many researchers have also mapped the growth habit-related traits in peanuts. Fonceka et al. (2012) detected 14 QTLs for plant type using chromosome segment substitution lines (CSSLs) derived from a cross between the wild synthetic allotetraploid (*A. ipaënsis* × *A. duranensis*)^4×^ and the cultivated “Fleur11.” Kayam et al. (2017) used an F_2:3_ population derived from Virginia-type peanut cultivars with spreading and bunch growth habit and found a single QTL on chromosome B05. Shirasawa et al. (2012) reported three QTLs for MSH with 4.80–19.20% phenotypic variation explained (PVE) and two QTLs for LBL with 14.2–21.1% PVE using F_2_ populations. Huang et al. (2015) detected three QTLs for MSH on A3, B4, and B7 in an F_2:3_ population. Li Y. et al. (2017) detected 11 QTLs for MSH and 16 QTLs for LBL and the QTL, ARS376-SEQ4G02, on linkage group 14 (LG14) influenced both traits. Lv et al. (2018) developed two different genetic background recombinant inbred line (RIL) populations to detect possible consistent QTLs for plant height and eventually found the QTLs on A09, B03, and B04.

With respect to domestication, the biggest change from wild ancestors to cultivated peanut was the transition of growth habit from prostrate growth to the erect (Muñoz et al., 2017). “Jihua 5” is an erect growth habit, while the “M130” is a spreading-type. A RIL population containing 188 individuals was developed from a cross of “Jihua 5” and “M130.” Therefore, the main objectives of this study were to develop a high-density genetic linkage map using SLAF-seq technology and identify QTLs controlling the growth habit in peanuts.

## Materials and Methods

### Plant Materials

**FIGURE 1:**
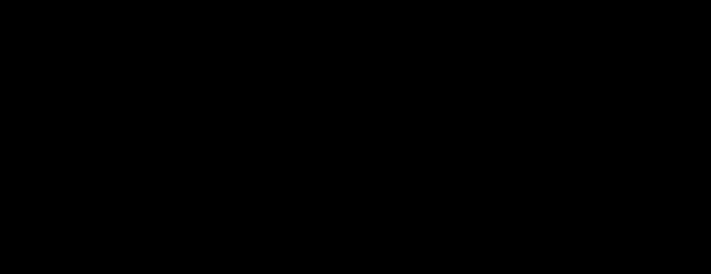
The different phenotypes in the parents. **(A)** The female parent “Jihua 5” with scale bar of 10 cm. **(B)** The male parent “M130” with scale bar of 10 cm.

A cross between “Jihua 5” and “M130” was used to develop an F_8_ population of 321 RILs using single seed decent (SSD) method. “Jihua 5” is an erect growth habit peanut genotype with a lateral branch angle (LBA) around 40°. “M130,” a spreading-type, has a LBA of around 90° (Figure 1). The 188 RILs were randomly selected from 321 individuals and their parents were planted in seven different environments. They were grown in Hai-Nan (HN) experimental station (N18°59′ and E109°11′) and Bao-Ding (BD) experimental station (N38°40′ and E115°30′) in 2016, which were referred to as 16HN and 16BD, respectively. In 2017, the experiments were conducted in Han-Dan (HD) station (N35°57′ and E115°09′) and BD, which were referred to as 17HD and 17BD, respectively. During 2018 growing seasons, the parents and population were planted in Tang-Shan (TS) station (N39°99′ and E118°70′), HD, and BD, which were referred to as 18TS, 18HD, and 18BD, respectively. Each plot contained one row with 1.5 m length, and the distance between each plant was around 17 cm, with a row spacing of 50 cm. Approximately 10 plants were planted in each row. Field management was performed under normal conditions. A randomized complete block design with two replicates was adopted in these four locations.

### Trait Measurements and Data Analysis

Three typical plants from each plot were harvested at the mature stage for LBA, MSH, LBL, extent radius (ER), and IOPT measurements. The LBA is defined as the angle between the main stem and the first lateral branch (Supplementary Figure S1). The MSH is the length of internode from the meristematic place of the first pair of the lateral branch on the main stem to parietal lobe (Supplementary Figure S1). The LBL is the length from the junction with the main stem to parietal lobe of the longest first lateral branch (Supplementary Figure S1). The ER is the longest distance between the main stem and the first lateral branch (Supplementary Figure S1). The IOPT is the ratio of the longest branch of the first pair lateral branches to MSH. The LBA was measured using an electronic protractor. The other traits were measured using a measuring tape.

The broad-sense heritability for growth habit-related traits in seven environments was calculated using the equation *h*^2^ = σ_g_^2^/(σ_g_^2^ + σ_ge_^2^/*n* + σ_e_^2^/*nr*), where σ_g_^2^, σ_ge_^2^, and σ_e_^2^ represent the genetic variance component, genotype–environment interaction variance component, and the random error variance component, respectively. The *n* and *r* were defined as the number of environments and replications, respectively. The SPSS 25.0 was used for the analysis of each variance component, variation coefficient, and descriptive statistics (IBM Corp, 2017).

### DNA Extraction and SLAF Library Construction

Genomic DNA was extracted from young leaf tissues of each sample using TaKaRa MiniBEST Plant Genomic DNA Extraction kit (TaKaRa, Dalian) and SLAF-seq was used to genotype a total of 188 individuals and the two parents, as previously described (Sun et al., 2013), with a few modifications. An improved SLAF-seq strategy was utilized in the experiment. Firstly, the reference genome of “Tifrunner” was used to design marker discovery experiments by simulating *in silico* the number of markers produced by different enzymes (Bertioli et al., 2019). Next, a SLAF pilot experiment was performed, and the SLAF library was conducted in accordance with the predesigned scheme. For the RIL population, genomic DNA was digested by two enzymes [Hpy166II and EcoRV-HF^®^, New England Biolabs (NEB), United States]. A single nucleotide (A) overhang was added subsequently to the digested fragments using Klenow fragment (3′→5′ exo^−^) (NEB) and dATP at 37°C. The duplex tag-labeled sequencing adapters (PAGE-purified, Life Technologies, United States) were then ligated to the A-tailed fragments using T4 DNA ligase. Polymerase chain reaction (PCR) was performed using the diluted restriction–ligation DNA samples, dNTP, Q5^®^ high-fidelity DNA polymerase, and PCR primers (forward primer: 5′-AATGATACGGCGACCACCGA-3′, reverse primer: 5′-CAAGCAGAAGACGGCATACG-3′) (PAGE-purified, Life Technologies, United States). The PCR products were then purified using Agencourt AMPure XP beads (Beckman Coulter, High Wycombe, United Kingdom) and pooled. The pooled samples were separated by 2% agarose gel electrophoresis. Fragments ranging from 364 to 414 bp (with indexes and adaptors) in size were excised and purified using a QIA Quick Gel Extraction Kit (Qiagen, Hilden, Germany). Gel-purified products were then diluted. Paired-end sequencing with reads length 125 bp was performed using an Illumina HiSeq 2500 system (Illumina, Inc., San Diego, CA, United States) according to the manufacturer’s recommendations.

### Sequence Data Grouping and Genotyping

Specific length amplified fragment marker identification and SNP genotyping were performed using procedures described by Sun et al. (2013). Low-quality reads (quality score < 20e) were removed and then raw reads were sorted to188 individuals according to duplex barcode sequences. After the barcodes and the terminal 5-bp positions were trimmed from each high-quality read, clean reads from the same sample were mapped onto the “Tifrunner” genome sequence using SOAP software (Li et al., 2008). The reads mapping on the same position were defined as one SLAF locus (Zhang et al., 2015). SNP loci of each SLAF locus were then detected between parents using the software GATK (DePristo et al., 2011), and SLAFs with more than three SNPs were filtered out first. Alleles of each SLAF locus were then defined according to parental reads with sequence depth >40-fold, while for each offspring the reads with sequence depth >12.37-fold were used to define alleles. The SLAF loci with more than four alleles were defined as repetitive SLAFs and subsequently discarded. Only SLAFs with two to four alleles were identified as polymorphic and considered potential markers. All polymorphism SLAFs loci were genotyped with consistency in the parental and offspring SNP loci. Polymorphic markers were classified into eight segregation patterns (ab × cd, ef × eg, hk × hk, lm × ll, nn × np, aa × bb, ab × cc, and cc × ab). Only the aa × bb pattern markers were used for construction of a linkage map.

### Linkage Map Construction

In order to reduce the genotyping errors and deletions due to the next-generation sequencing, high map strategy was used to construct a high-density linkage map for the RIL population (Liu et al., 2014). All high quality of SLAF markers were distributed into 20 LGs based on their locations on chromosomes. A detailed MST map algorithm was used to order SLAF markers (Wu et al., 2008) and the SMOOTH algorithm (van Os et al., 2005) was used to correct genotyping errors following marker ordering. The four procedures were followed to construct all LGs: (1) the SLAF markers were partitioned into LGs using a single-linkage clustering algorithm based on a pairwise modified independent LOD score for the recombination frequency; (2) using a combination of Gibbs sampling, spatial sampling, and the simulated annealing algorithm, the ordering module sequenced SLAF markers, and estimated linkage distances; (3) the error correction strategy of SMOOTH was then conducted according to parental contribution of genotypes (van Os et al., 2005), and a *k*-nearest neighbor algorithm was applied to impute missing genotypes (Huang et al., 2012). Skewed markers were then added into this map by applying a multi-point method of maximum likelihood; and (4) map distances were estimated using the Kosambi mapping function (Kosambi, 1944).

### QTL Analysis and Candidate Genes Prediction for Hot Spot Regions

Quantitative trait locus IciMapping version 4.0 (Wang et al., 2014) was performed to identify the QTLs of each trait in different environments by using inclusive composite interval mapping of additive (ICIM-ADD). To claim significant QTLs, the LOD score was set at 2.5 to detect significant QTLs. QTLs were named with initial letter “q” followed by the trait name and LG. A numeric was added if two or more QTLs were identified in the same LG. For example, two QTLs for LBA were detected on LGB04, and then they were named *qLBAB04.1* and *qLBAB04.2*. The QTLs with more than 10% PVE were considered as major QTLs, while other QTLs were considered as minor QTLs (Luo et al., 2017). The MapChart 2.3 software (Voorrips, 2002) was used to graph the genetic linkage map and the QTLs. QTLs of the growth habit-related traits co-localized in the same genomic region were defined as the hot spot region. The genes in the hot spot regions were predicted as candidates based on the “Tifrunner” genome sequence (Bertioli et al., 2019). The functional annotations from the Gene Ontology (GO), the Kyoto Encyclopedia of Genes and Genomes (KEGG), and the Non-Redundant Protein Database (NR) at NCBI were extracted to categorize the candidate genes.

## Results

### Phenotypic Variation of Growth Habit-Related Traits in RILs and Their Parents

**FIGURE 2:**
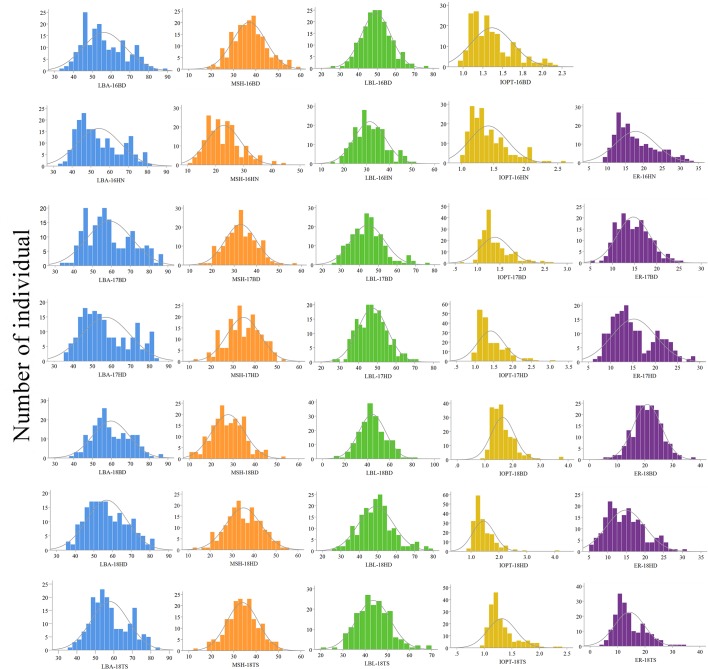
Phenotypic distributions of growth habit-related traits in the RIL population under seven environments.

The parents “Jihua 5” and “M130” showed distinct differences in LBA, MSH, LBL, ER, and IOPT in all environments (Table 1 and Supplementary Figure S2). Large variations and transgressive segregations were observed in RILs in the seven environments for all traits except IOPT in 16BD, 18BD, and 18TS (Table 1). Continuous distributions were observed in Figure 2 and the Shapiro–Wilk (*w*) test indicated that the phenotypic data of MSH and LBL were normally distributed except in 16HN, 17BD, and 18HD. The data of ER were normally distributed in 18BD, but not for other locations. The genotypic coefficient of variation (GCV) and broad-sense heritability of measured traits ranged from 6.60 to 22.32% and 86 to 92%, respectively (Table 2). The ANOVA results indicated significant differences in genotype, environment, and genotype × environment for all traits except genotype × environment for LBA (Table 2). The LBA had a significant positive relationship with ER (*r* = 0.843) and IOPT (*r* = 0.689), while LBA had a significant negative association with MSH (*r* = −0.384) (Table 3). MSH and ER were also negatively correlated, with a correlation coefficient of −0.283 in the environment of 17HD, whereas MSH had a significant positive correlation with LBL (*r* = 0.681) (Table 3).

**Table 1:** Phenotypic variation for five traits of the RILs and their parents in seven environments.

Env	Trait	Parents		RILs population							
		P1	P2	Max	Min	Mean	*SD*	*cv* (%)	Shapiro–Wilk (*w*)	Kurt	Skew
16BD	LBA	45.80	81.03	87.80	34.10	56.36	11.46	20.33	0.96 (<0.01)	−0.64	0.46
	MSH	45.50	23.17	59.75	19.00	37.05	7.43	20.05	0.99 (0.30)	0.11	0.31
	LBL	49.67	61.83	75.25	25.00	49.35	7.92	16.05	0.99 (0.82)	0.31	0.18
	IOPT	1.09	2.67	2.18	0.98	1.36	0.26	19.12	0.92 (<0.01)	0.52	1.01
16HN	LBA	37.50	77.00	79.88	34.30	54.08	11.35	20.99	0.94 (<0.01)	−0.81	0.55
	MSH	35.50	17.13	44.00	10.63	22.84	6.04	26.44	0.96 (<0.01)	0.52	0.73
	LBL	38.88	36.88	50.38	15.33	31.38	6.89	21.96	0.99 (0.054)	−0.11	0.39
	ER	17.13	31.75	32.50	9.17	17.79	5.54	31.14	0.93 (<0.01)	−0.30	0.78
	IOPT	1.10	2.15	2.62	0.98	1.42	0.30	21.13	0.90 (<0.01)	1.49	1.24
17BD	LBA	43.60	83.23	87.25	33.70	58.90	12.12	20.58	0.97 (<0.01)	−0.72	0.39
	MSH	38.40	19.17	56.26	15.95	32.88	7.00	21.29	0.99 (0.72)	0.08	0.19
	LBL	40.63	46.60	75.90	26.28	44.37	8.94	20.15	0.97 (<0.01)	0.84	0.71
	ER	8.37	21.03	25.70	5.00	14.72	3.68	25.00	0.99 (<0.05)	−0.15	0.38
	IOPT	1.06	2.43	2.69	0.67	1.39	0.33	23.74	0.88 (<0.01)	2.46	1.45
17HD	LBA	39.02	79.10	83.91	35.59	57.15	12.65	22.13	0.94 (<0.01)	−0.90	0.48
	MSH	45.42	25.15	52.28	13.73	34.81	7.57	21.75	0.99 (0.26)	−0.52	−0.11
	LBL	48.68	55.03	70.20	27.33	47.50	7.93	16.69	0.99 (0.28)	−0.01	0.31
	ER	10.92	19.93	28.70	6.60	15.21	4.92	32.35	0.95 (<0.01)	−0.55	0.60
	IOPT	1.07	2.19	3.02	0.98	1.42	0.35	24.65	0.84 (<0.01)	2.97	1.61
18BD	LBA	37.50	72.77	86.13	39.10	59.72	9.71	16.26	0.98 (<0.01)	−0.57	0.30
	MSH	38.00	19.17	53.00	10.17	27.75	7.55	27.21	0.99 (0.39)	0.09	0.27
	LBL	40.00	76.17	80.00	13.00	44.39	11.96	26.94	0.99 (0.63)	0.15	0.21
	ER	10.33	34.17	36.83	5.83	20.88	5.11	24.47	0.99 (0.99)	0.25	−0.03
	IOPT	1.05	3.97	3.83	0.91	1.64	0.42	25.61	0.86 (<0.01)	6.29	1.93
18HD	LBA	45.80	81.03	81.35	35.00	56.87	10.71	18.83	0.98 (<0.05)	−0.67	0.32
	MSH	45.50	23.17	55.67	12.83	35.16	7.98	22.70	0.99 (0.84)	−0.35	0.02
	LBL	49.67	61.83	78.75	25.25	49.01	9.47	19.32	0.98 (<0.05)	0.62	0.43
	ER	7.67	20.17	30.50	5.00	14.80	5.16	34.86	0.97 (<0.01)	−0.13	0.54
	IOPT	1.09	2.67	4.14	0.94	1.45	0.39	26.90	0.80 (<0.01)	12.65	2.65
18TS	LBA	47.40	78.37	83.40	36.20	57.70	10.22	17.71	0.97 (<0.01)	−0.68	0.35
	MSH	45.50	24.83	50.33	13.00	34.00	6.99	20.56	0.99(0.80)	−0.03	−0.07
	LBL	51.67	60.67	69.88	22.00	43.71	7.69	17.59	0.99 (0.054)	0.76	0.42
	ER	10.50	23.13	34.50	4.75	14.38	5.42	37.69	0.93 (<0.01)	1.02	1.04
	IOPT	1.14	2.44	2.36	0.94	1.32	0.26	19.70	0.85 (<0.01)	2.40	1.55

*P1, female parent Jihua5; P2, male parent M130. Env. is the environment. The unit of measurement for LBA is degree. The unit of measurement for MSH, LBL, and ER is centimeter. Min, minimum; Max, maximum; SD, standard deviation; Skew, skewness; Kurt, kurtosis; cv is the coefficient of variation.*

**Table 2:** Analysis of variance for five traits in the RIL population under seven environments.

Trait	Variables	*df*	Mean square	*F*-value	*P*-value	GCV%	*h*^2^
LBA	RILs	187	1,557.91	3.92	<0.01	17.17	0.86
	Environments	6	1,269.34	3.20	<0.01		
	RILs × environments	1,121	27.23	0.07	1		
	Error	1,300	397.05				
MSH	RILs	187	495.04	73.09	<0.01	17.96	0.92
	Environments	6	9295.71	1,372.40	<0.01		
	RILs × environments	1,120	38.05	5.62	<0.01		
	Error	1,294	6.77				
LBL	RILs	187	680.60	72.19	<0.01	15.06	0.90
	Environments	6	1,3993.03	1,484.15	<0.01		
	RILs × environments	1,120	66.08	7.01	<0.01		
	Error	1,303	9.43				
ER	RILs	187	179.45	47.33	<0.01	22.32	0.89
	Environments	5	2,486.53	655.78	<0.01		
	RILs × environments	932	23.34	6.16	<0.01		
	Error	1,111	3.79				
IOPT	RILs	187	1.00	46.08	<0.01	6.60	0.91
	Environments	6	4.08	187.57	<0.01		
	RILs × environments	1,120	0.09	4.27	<0.01		
	Error	1,284	0.02				

*GCV is the genotypic coefficient of variation.*

**Table 3:** Correlation analysis of five traits in the RIL population under seven environments.

Environment	Trait	LBA	MSH	LBL	ER	IOPT
16BD	LBA	1				
	MSH	−0.269^∗∗^	1			
	LBL	0.337^∗∗^	0.524^∗∗^	1		
	ER	−	−	−	1	
	IOPT	0.609^∗∗^	−0.624^∗∗^	0.312^∗∗^	−	1
16HN	LBA	1				
	MSH	−0.116	1			
	LBL	0.448^∗∗^	0.681^∗∗^	1		
	ER	0.750^∗∗^	0.126	0.718^∗∗^	1	
	IOPT	0.643^∗∗^	−0.525^∗∗^	0.234^∗∗^	0.630^∗∗^	1
17BD	LBA	1				
	MSH	−0.272^∗∗^	1			
	LBL	0.397^∗∗^	0.455^∗∗^	1		
	ER	0.714^∗∗^	−0.63	0.584^∗∗^	1	
	IOPT	0.646^∗∗^	−0.558^∗∗^	0.449^∗∗^	0.619^∗∗^	1
17HD	LBA	1				
	MSH	−0.384^∗∗^	1			
	LBL	0.420^∗∗^	0.409^∗∗^	1		
	ER	0.843^∗∗^	−0.283^∗∗^	0.549^∗∗^	1	
	IOPT	−0.689^∗∗^	−0.689^∗∗^	0.337^∗∗^	0.681^∗∗^	1
18BD	LBA	1				
	MSH	−0.162^∗^	1			
	LBL	−0.297^∗∗^	0.664^∗∗^	1		
	ER	0.539^∗∗^	0.273^∗∗^	0.688^∗∗^	1	
	IOPT	0.584^∗∗^	−0.384^∗∗^	0.394^∗∗^	0.523^∗∗^	1
18HD	LBA	1				
	MSH	−0.216^∗∗^	1			
	LBL	0.447^∗∗^	0.489^∗∗^	1		
	ER	0.726^∗∗^	−0.093	0.528^∗∗^	1	
	IOPT	0.586^∗∗^	−0.600^∗∗^	0.327^∗∗^	0.537^∗∗^	1
18TS	LBA	1				
	MSH	−0.242^∗∗^	1			
	LBL	0.296^∗∗^	0.627^∗∗^	1		
	ER	0.680^∗∗^	−0.029	0.507^∗∗^	1	
	IOPT	0.612^∗∗^	−0.591^∗∗^	0.217^∗∗^	0.538^∗∗^	1

*^∗^Correlation is significant at the 0.05 level. ^∗∗^Correlation is significant at the 0.01 level.*

### High-Throughput SLAF Sequencing and Genotyping

A total of 472.94 GB of raw data containing 1,576.48 M reads were obtained for the SLAF library. The guanine–cytosine (GC) content was 39.13% in average and the Q30 ratio (quality score of at least 30, indicating a 1% chance of error) was 94.63%. For the paternal inbred line (“M130”), 1,021,241 SLAFs were generated from 50,864,965 reads with an average coverage of 48.83-fold for each SLAF. For the maternal line (“Jihua5”), 1,176,582 SLAFs were produced from 53,171,750 reads and the average coverage of each SLAF was 41.09-fold. For the analysis of the RIL mapping population, 604,234 SLAFs were screened from the 7,832,134 reads in each genotype with an average coverage of 12.37-fold (Table 4).

**Table 4:** Summary of SLAF numbers and marker depths.

Sample	Raw read numbers	SLAF numbers	SNP numbers	Homo-SNP numbers	Total depth (×)	Average depth (×)
M130	50,864,965	1,021,241	425,047	416,367	49,862,489	48.83
jihua5	53,171,750	1,176,582	463,462	451,890	48,348,687	41.09
RILs	7,832,134	604,234	302,464	294,721	7,447,985	12.37

**FIGURE 3:**
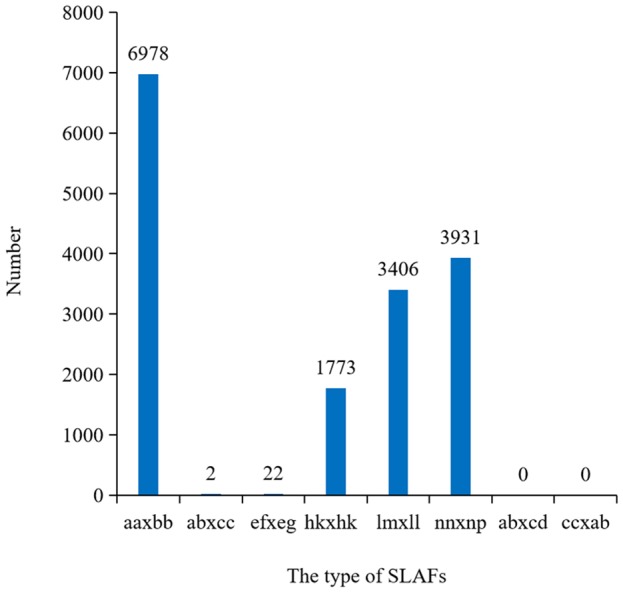
Numbers of SLAFs markers in each segregation types. aa × bb indicated the parents were homozygous; ef × eg, hk × hk, and ab × cd indicated the parents were heterozygosis; lm × ll, nn × np, ab × cc, and cc × ab indicated one of the parents was homozygous and the other was heterozygosis.

After filtering or discarding heterozygous SNPs, the same homozygous SNPs presented in both parents were removed. A total of 16,112 SNPs were identified and were successfully encoded and grouped into eight segregation patterns: ab × cd, ef × eg, ab × cc, cc × ab, hk × hk, lm × ll, nn × np, and aa × bb according to the genotype encoding rule. Only aa × bb segregation pattern of 6,978 SNPs was used for construction of the linkage map (Figure 3). Raw data have been submitted to the NCBI SRA database and the SRA accession number is PRJNA517739^1^.

### High-Density Genetic Map Construction

**FIGURE 4:**
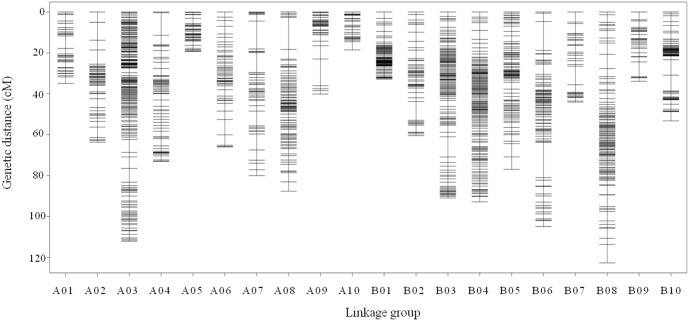
High density genetic map with the 2,808 SNP markers distributed on 20 LGs of peanut. The *x*-axis represents the number of LGs and the *y*-axis represents the marker’s location in genetic map.

All 2,808 high-quality markers were successfully assigned onto 20 LGs compared with the reference genome (Figure 4). The final molecular linkage map was 1,308.20 cM in total length with an average inter-marker distance of 0.47 cM (Table 5). B08 was the largest LG containing 300 markers, 122.60 cM in length, and an average distance of 0.41 cM. B07 was the smallest LG, which only had 39 markers, with the length of 44.07 cM and an average distance of 1.16 cM. The largest gap of linkage between markers was 17.1 cM located in B06. The gap with <5 cM was counted for 98.50% on the linkage map (Table 5).

**Table 5:** Basic characteristics of the 20 LGs constructed from the RIL population.

Linkage group	Total marker	Total distance (cM)	Average distance (cM)	Max gap (cM)	Gap < 5 cM (%)
A01	49	34.93	0.73	6.09	97.92
A02	249	63.67	0.26	8.74	98.39
A03	247	112.10	0.46	6.76	98.78
A04	86	73.03	0.86	11.00	98.82
A05	220	19.36	0.09	2.62	100.00
A06	251	65.97	0.26	7.51	99.20
A07	109	80.01	0.74	13.60	95.37
A08	141	87.64	0.63	15.60	99.29
A09	48	40.13	0.85	13.00	95.74
A10	40	18.54	0.48	3.99	100.00
B01	197	32.85	0.17	3.22	100.00
B02	71	60.43	0.86	9.16	98.57
B03	134	90.94	0.68	9.85	99.25
B04	155	92.83	0.60	4.35	100.00
B05	91	76.93	0.85	6.53	97.78
B06	243	104.90	0.43	17.10	99.17
B07	39	44.07	1.16	9.07	97.37
B08	300	122.60	0.41	8.99	98.66
B09	55	33.89	0.63	7.25	98.15
B10	83	53.24	0.65	7.73	97.56
Total	2,808	1,308.20	0.47	/	98.50

### Visualization and Evaluation of the Genetic Map

The haplotype and heat maps were used to assess the quality of the peanut genetic map. Haplotype map reflected the ratio of double crossover for the population and implied the genotyping errors or hot spots region with genomic recombination. The higher the ratio of double crossovers, the more problems exist in the genotyping and marker ordering. The haplotype maps were generated for each individual of the 188 RILs as well as the parental lines using 2,808 markers. As shown in Supplementary Figure S3, there were a few double crossovers or deletions in the 20 LGs indicating that the genetic map was of high quality. The heat maps were used to analyze and evaluate the frequency of recombination between markers from each LG. The heat maps also showed that most LGs were of high quality (Supplementary Figure S4). The collinearity analysis was performed between the genetic map and the peanut reference genome (Tifrunner), and all of the SLAF markers were anchored to the peanut reference genome. The results from the analysis showed relatively high collinearity between LGs and corresponding chromosomes (Supplementary Figure S5).

### QTL Mapping for Growth Habit-Related Traits

**FIGURE 5:**
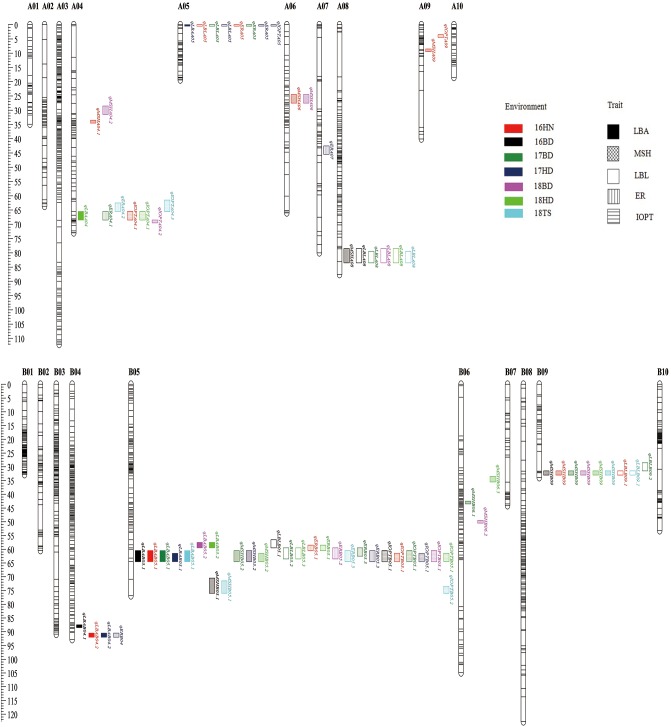
The distribution of QTLs for growth habit-related traits on the genetic linkage map.

A total of 39 QTLs associated with growth habit-related traits were detected using ICIM-ADD based on IciMapping (Supplementary Table S1). For LBA, a total of six QTLs were identified and explained 5.02–21.87% of the phenotypic variance. *qLBAB05.1* has the largest additive effect QTL (*a* = −5.80 ) and contributes 21.87% to PVE in 17HD. Another major QTL was *qLBAB05.2* located on B05 and detected in two environments. The minor QTLs (*qLBA04.1*, *qLBAB04.2*, *qLBAA04*, and *qLBAA05*) were mapped on A04, A05, and B04 and can explain 5.02–7.85% of the phenotypic variance, respectively (Supplementary Table S1). For ER and IOPT, eight and seven QTLs were detected, respectively. Among them, the two most significant QTLs (*qERB05.1* and *qIOPTB05.1*) were mapped on B05. One minor QTL (*qERA05*) related to ER trait on A05 was identified in three environments (16HN, 17BD, and 17HD) (Supplementary Table S1). The QTLs *qIOPTB05.1* and *qIOPTB05.3* located on B05 were the most significant for IOPT with about 14% PVE, while one minor QTL (*qIOPTA04.1*) associated with IOPT on A04 was detected in two environments (16HN and 18HD) (Supplementary Table S1). A total of 11 QTLs for MSH were detected and distributed on seven chromosomes, with the PVE of 5.67–17.68% and the additive effects of −2.24 to 3.11. Among these QTLs, one QTL (*qMSHB09*) on B09 was consistently detected in six out of a total of seven environments with the PVE of 5.67–13.73%. The QTL (*qMSHB05.2*) located on B05 in a single environment (17HD) can explain 17.68% of the phenotypic variance (Supplementary Table S1). For LBL, seven QTLs (*qLBLA08*, *qLBLB05.1*, *qLBLB05.2*, *qLBLB05.3*, *qLBLA05*, *qLBLB09.1*, and *qLBLB09.2*) were detected in various environments (Supplementary Table S1). It is interesting that some major QTLs were common loci for the different traits. For example, the QTLs *qLBAB05.1*, *qMSHB05.2*, *qLBLB05.2*, *qLBLB05.3*, *qERB05.3*, and *qIOPTB05.1* were co-localized on B05 on the region of 60.50–64.50 cM underlying LBA, MSH, LBL, ER, and IOPT (Figure 5). Similarly, the QTLs found on A04, A05, A08, and B09 can control two or more traits (Figure 5).

### Candidate Genes Identification for Hot Region on B05

Twelve important QTLs associated with peanut growth habit-related traits on B05 were verified and mapped the flanking markers on the 168,048 bp physical region from 159,819,755 to 159,987,803 bp of cultivated peanut physical genome (Supplementary Dataset S1), in which 19 putative genes were annotated. By GO annotation, there were 12 genes assigned at least one GO term. The 12 genes were divided into three GO categories, namely “cellular component” (15), “molecular function” (2), and “biological process” (25). These genes were involved in the biological processes: cytokinin-activated signaling pathway (GO:0009736); positive regulation of long-day photoperiodism (GO:0048578); responding to blue light (GO:0009637); responding to far red light (GO:0010218); responding to light intensity (GO:0009642); and temperature compensation of the circadian clock. These genes have highly promising functions of regulating the growth habit (Supplementary Dataset S2). Through KEGG analyses, *SZWP5S.1* and *SZWP5S.2* encoding GIGANTEA-like protein were assigned to circadian rhythm-plant pathways. Using the NR database, the number of 6, 12, and 1 homologous genes were identified from *Glycine soja*, *Glycine max*, and *Cicer arietinum*, respectively.

## Discussion

A high-density genetic linkage map plays a crucial role in many fields of fundamental and applied research, such as map-based cloning of genes, whole-genome sequencing for an assembly sequence, marker-assisted breeding, and QTL analysis. The first peanut SSR-based genetic map was developed with 135 loci onto 22 LGs using a RIL population (Varshney et al., 2009a). Subsequently, more SSR markers have been developed, and more SSR-based maps have been constructed (Hong et al., 2008, 2010; Huang et al., 2015). Although such SSR markers are available to construct the genetic map, the density of the map was restricted. The development of high-throughput sequencing technologies and low price are the prerequisites for detecting abundant SNPs markers throughout the genome. SLAF-seq, a relatively efficient experimental method, is different from others in that sequence-specific restriction fragment length was sequenced with better repeatability and accuracy, rather than sequencing random restriction fragments, like RAD-seq. Since the reference genome sequences of two diploid ancestors, *A. duranensis* (AA, 2*n* = 2*x* = 20) and *A*. *ipaensis* (BB, 2*n* = 2*x* = 20), were published, some researchers used the peanut diploid reference genome to genotype the SNP markers and to perform the collinearity analyses by comparing the positions of markers between the genetic and physical maps (Gupta et al., 2015; Agarwal et al., 2018; Hu et al., 2018; Wang et al., 2018). They also utilized the diploid reference to detect candidate genes in the QTL regions. Although the reference genome sequences of *A. duranensis* and *A. ipaensis* provided important genomic information of peanut, all the cultivated peanut cultivars are allotetraploids (Bertioli et al., 2016). Hence, the genome sequence of allotetraploids peanut ensures the accuracy of genetic map construction and candidate genes identification. The reference genome of allotetraploids peanut “Tifrunner” (Bertioli et al., 2019) was used in this study and constructed the linkage map that contains 2,808 SNP markers with the average distance of inter-marker of 0.47 cM. To our knowledge, the high density of markers in this map is sufficient to detect QTLs.

A total of 39 QTLs for LBA, MSH, LBL, ER, and IOPT distributed on 10 chromosomes were identified across the seven environments by IciMapping 4.0 software, with LOD scores ranging from 2.57 to 13.94, and 4.55 to 27.74% of the PVE (Figure 5 and Supplementary Table S1). Growth habit is a complex and comprehensive trait highly influenced by the domestication process. In the study, rather than using naked eyes, the angle between the main stem and the first lateral branch, the ER, and the IOPT was measured to represent the classification of the plant type. Interestingly, some QTLs (*qLBAB05.1*, *qLBAB05.2*, *qERB05.1*, *qERB05.2*, *qERB05.3*, *qIOPTB05.1*, and *qIOPTB05.2*) were mapped in the common genome region on B05 for all of these three traits across different environments, spanning approximately 0.17 Mb physical interval between 1,59,819,755 and 1,59,987,803. This physical position is different from the previous report on B05 (1,45,553,897–1,46,649,943) (Kayam et al., 2017). MSH is an important agronomic trait affecting photosynthesis efficiency, lodging, and yield. The first LBL is also an important trait for peanut breeding because it can affect planting density and yield. Both of these are quantitative traits. Previous studies showed that MSH and LBL were positively correlated. However, no consistent QTLs from different populations were detected for MSH or LBL because these QTLs could be specific for the populations (Shirasawa et al., 2012; Huang et al., 2015; Li Y. et al., 2017). In this study, the data showed a strong significant positive correction (*r* = 0.681) between MSH and LBL (Table 3). Eleven novel QTLs on A04, A06, A09, B05, B06, B08, and B09 with 5.67–17.68% PVE for MSH and 6 QTLs on A05, A08, B05, and B09 with 5.90–10.05% PVE for LBL were mapped. It was noteworthy that *qMSHB05.2*, *qLBLB05.1*, and *qLBLB05.2* were co-localized on B05. The region from 57.50 to 64.50 cM on B05 was the hot QTL spot for all growth habit-related traits (LBA, MSH, LBL, ER, and IOPT), suggesting that multiple genes or genes with pleiotropic effects control these traits. This also can be verified by the significant correlation between these traits. For these overlapping QTLs, a positive additive effect indicated the allele derived from the female parent “Jihua 5” improving the value of MSH, while a negative additive effect indicated that the allele for increasing the value of LBA, ER, IOPT, and LBL came from the male parent “M130.”

The evolved architectural traits during domestication include the extent of the vegetative shoot and inflorescence branching, branch angle, as well as internode elongation (Teichmann and Muhr, 2015). In this study, a total of 19 candidate genes for growth habit were identified on the co-localized genome region on B05, which spanned 0.17 Mb physical intervals and 19 genes were annotated by at least one category of either GO, KEGG, or NR databases. GO annotations indicated that *SZWP5S.1* and *SZWP5S.2* play a role in light signal functions. Meanwhile, through KEGG analysis, *SZWP5S.1* and *SZWP5S.2* are predicted to have a role in the circadian rhythm-plant pathway. *H1TMWI.1* and *H1TMWI.2* have a role in a cytokinin-activated signaling pathway responding to light intensity. One strategy for improving yield is to increase planting density; however, a key factor that limits planting density in the practical application of agriculture is the plant’s shade-avoidance response. The shade-avoidance syndrome consists of increased plant height and reduced branching and early flowering that are triggered by changes in the quality of light (Casal, 2012; Xie et al., 2017). Elongation of the stem is increased under shade conditions because of the increased free indole acetic acid (IAA) due to the competition for the light with neighboring plants (Gallavotti, 2013). Annotations in the NR database implied that six genes (*FXXJ78.1*, *IS4878.1*, *IS4878.3*, *FXXJ78.2*, *H1TMWI.1*, and *IS4878.2*) were related to tryptophan aminotransferase-related protein 4 (*G. soja*). Tryptophan aminotransferase which is encoded by the TAA gene converts tryptophan into indole pyruvic acid (IPA) and subsequently converts IPA into IAA by monooxygenase enzymes which are encoded by the *YUCCA* gene family (Stepanova et al., 2008; Tao et al., 2008; Mashiguchi et al., 2011; Won et al., 2011). IAA is a common auxin which can affect plant growth and development. These genes, which are involved in light and hormones, need to be verified. The annotated genes could contribute to a better understanding of the genetic mechanism behind the growth habit-related traits. Although the plant type is visible, it is time-consuming for breeders to assess the performances of genotypes in changeable environments. Therefore, it is necessary to screen the markers linked with the growth habit-related traits to improve breeding efficiency. These presumptive genes could provide a potential for MAS in peanut breeding for growth habit-related traits.

## Conclusion

A high-density genetic map of cultivated peanuts was constructed using SLAF-seq techniques. It consists of 2,808 markers on the 20 LGs with a total length of 1,308.20 cM and an average inter-marker distance of 0.47 cM. A total of 39 QTLs associated with growth habit-related traits were identified using a RIL population under seven environments in four locations. Furthermore, multiple QTLs were co-localized on the chromosome B05 under at least two environments. The identified genomic regions and putative genes that involved light and hormones will support the further development of new strategies at the molecular genetic level to promote the application of MAS in peanut breeding for improved growth habit.

## Data Availability

The datasets generated for this study can be found in the NCBI SRA database and the SRA accession number is PRJNA517739 (https://www.ncbi.nlm.nih.gov/sra/PRJNA517739).

## Author Contributions

LFL and CC designed the experiments. XY, SC, and MJH developed the mapping population. LL, XM, and MYH performed the growth habit-related traits measurements of the plant materials. LL and XLY constructed the genetic linkage map and performed QTL analysis. LL analyzed the result and wrote the manuscript. LFL, CC, GM, and HZ revised the manuscript. All authors contributed to the manuscript and approved the final version of the manuscript to be published.

## Conflict of Interest Statement

The authors declare that the research was conducted in the absence of any commercial or financial relationships that could be construed as a potential conflict of interest.

## Footnotes

1 https://www.ncbi.nlm.nih.gov/Traces/study/?acc=PRJNA517739
