# Supplementary material for: Construction of High-Density Genetic Map and Mapping Quantitative Trait Loci for Growth Habit-Related Traits of Peanut (Arachis hypogaea L.)
Source: Front Plant Sci. 2019 Jun 12;10:745. doi: 10.3389/fpls.2019.00745 (PMC6584813; doi:10.3389/fpls.2019.00745)
Supplement: Supplementary file 1 [file Image_1.pdf]

Supplementary Figure S1. The model for description of growth habit related traits in peanut.

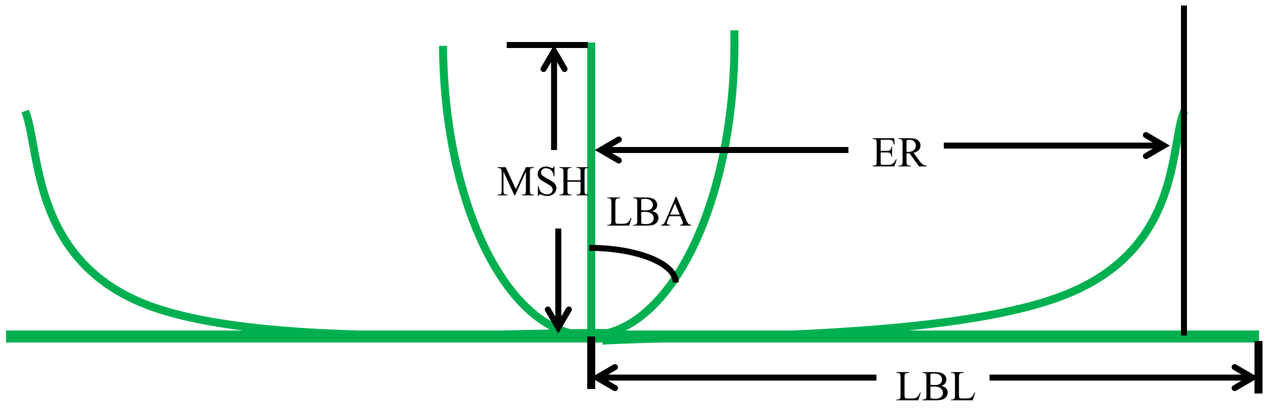

LBA, lateral branch angle, the angle between main stem and the first lateral branch. MSH, main stem height, the length of internode from meristematic place of the first pair of the lateral branch on the main stem to parietal lobe. LBL, lateral branch length, the length from the junction with main stem to parietal lobe of the longest first lateral branch. ER, extent radius, the longest distance between main stem and the first lateral branch.
