# Supplementary material for: Construction of High-Density Genetic Map and Mapping Quantitative Trait Loci for Growth Habit-Related Traits of Peanut (Arachis hypogaea L.)
Source: Front Plant Sci. 2019 Jun 12;10:745. doi: 10.3389/fpls.2019.00745 (PMC6584813; doi:10.3389/fpls.2019.00745)

Supplementary Figure S5. The correlation of the genetic and physical positions. Axis of abscissa represents the genetic linkage groups and axis of ordinate represents the physical positions.

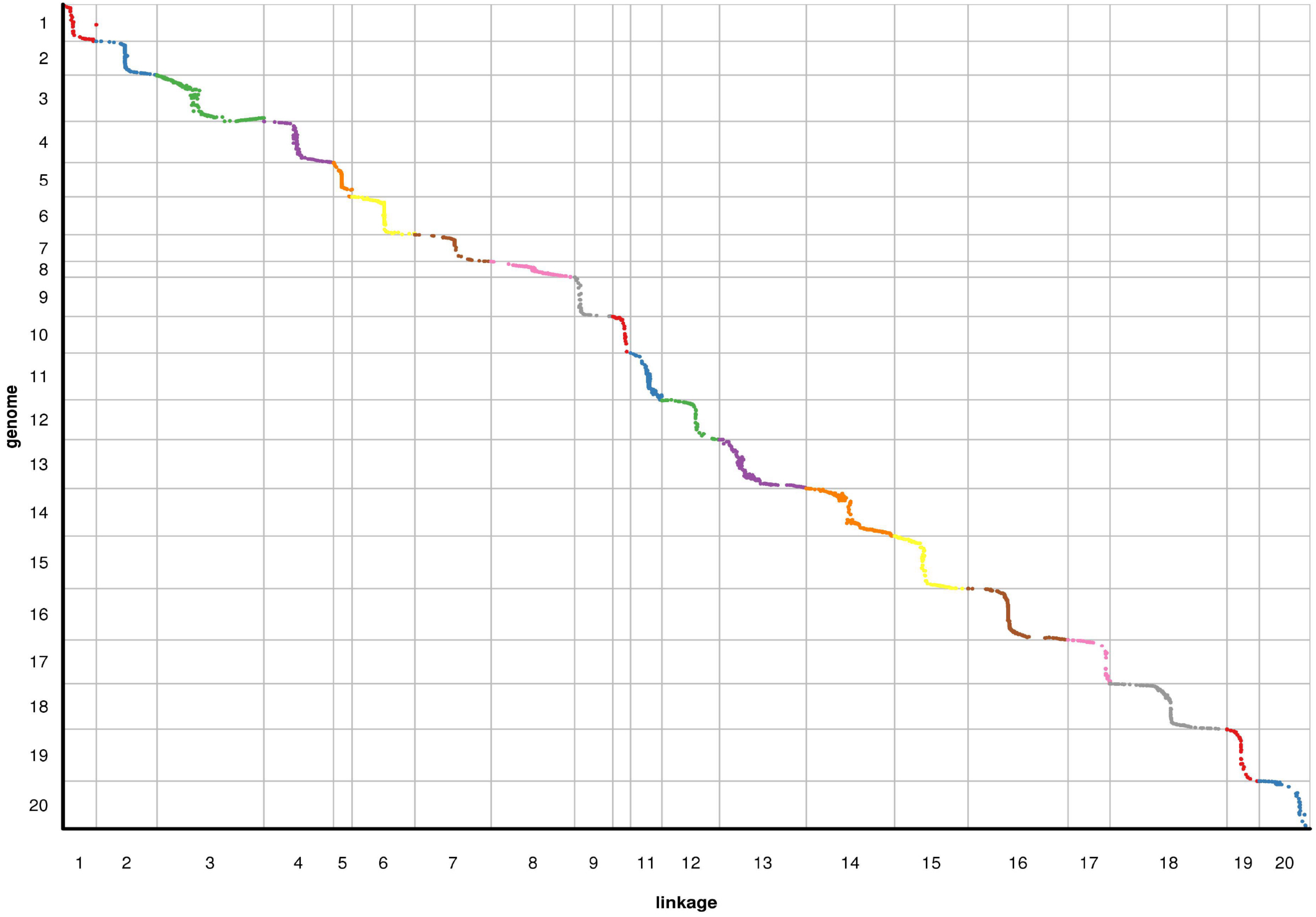

Supplement: Supplementary file 3 [file Image_5.pdf]
