# Supplementary figures and images for: Construction of High-Density Genetic Map and Mapping Quantitative Trait Loci for Growth Habit-Related Traits of Peanut (Arachis hypogaea L.)
Source: Front Plant Sci. 2019 Jun 12;10:745. doi: 10.3389/fpls.2019.00745 (PMC6584813; doi:10.3389/fpls.2019.00745)

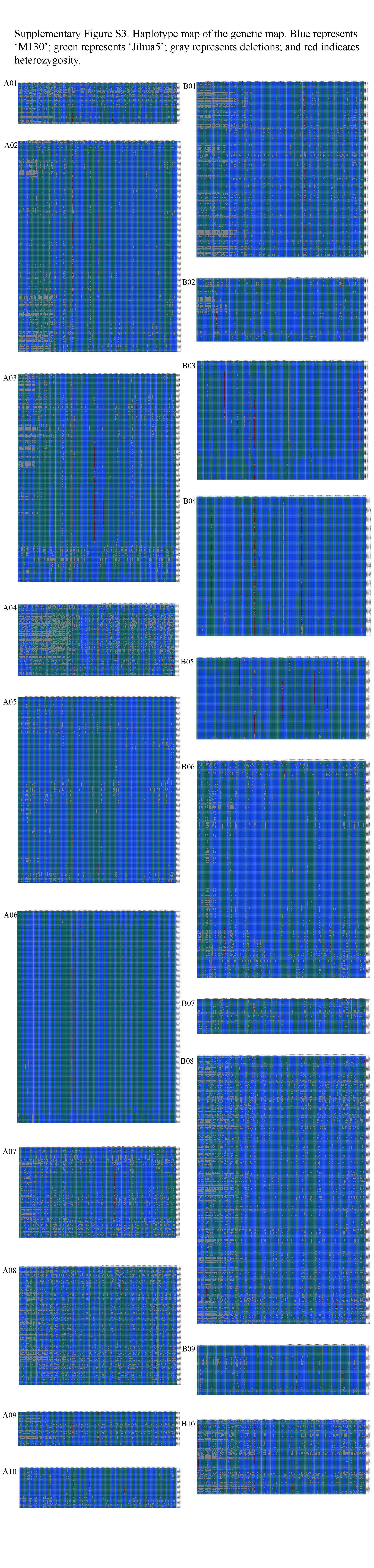

Supplement: Supplementary file 4 [file Image_3.jpg]

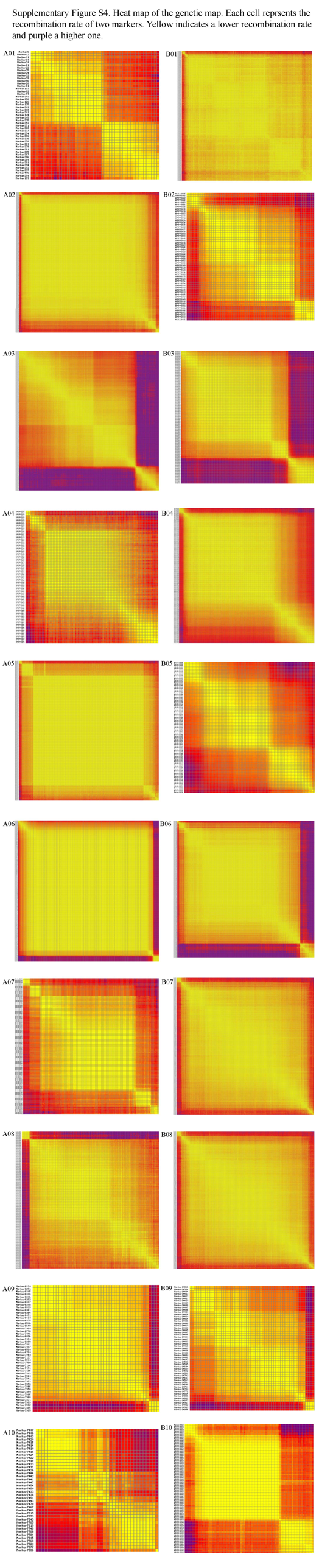

Supplement: Supplementary file 5 [file Image_4.jpg]
